# Short and long-term evaluation of the impact of proton minibeam radiation therapy on motor, emotional and cognitive functions

**DOI:** 10.1038/s41598-020-70371-w

**Published:** 2020-08-11

**Authors:** Charlotte Lamirault, Valérie Doyère, Marjorie Juchaux, Frederic Pouzoulet, Dalila Labiod, Remi Dendale, Annalisa Patriarca, Catherine Nauraye, Marine Le Dudal, Grégory Jouvion, David Hardy, Nicole El Massioui, Yolanda Prezado

**Affiliations:** 1 Translational Research Department, Experimental Radiotherapy Platform, Institut Curie, PSL Research University, Orsay, France; 2 Université Paris-Saclay, CNRS, Institut des Neurosciences Paris-Saclay, 91190 Gif-sur-Yvette, France; 3 Laboratoire de Physique des 2 Infinis Irène Joliot-Curie (IJCLab-UMR 9012), CNRS/Université Paris-Saclay/Université de Paris, Campus Universitaire, Orsay, France; 4 Radiation Oncology Department, Centre de Protonthérapie d’Orsay, 101, Institut Curie, PSL Research University, 91898 Orsay, France; 5 Institut Pasteur, Neuropathologie Expérimentale, 75015 Paris, France; 6 Ecole Nationale Vétérinaire d’Alfort, Biopôle, Unité d’Histologie, d’Embryologie et d’Anatomie Pathologique, Université Paris-Est, Maisons-Alfort, France; 7 Physiopathologie des Maladies Génétiques d’Expression Pédiatrique, Assistance Publique des Hôpitaux de Paris, Hôpital Armand-Trousseau, UF de Génétique Moléculaire, Sorbonne Université, INSERM, Paris, France; 8 Institut Curie, Inserm U 1021-CNRS UMR 3347, University Paris Saclay, PSL Research University, Bat 110, Campus d’Orsay, Orsay, France

**Keywords:** Preclinical research, Translational research

## Abstract

Radiotherapy (RT) is one of the most frequently used methods for cancer treatment. Despite remarkable advancements in RT techniquesthe treatment of radioresistant tumours (i.e. high-grade gliomas) is not yet satisfactory. Finding novel approaches less damaging for normal tissues is of utmost importance. This would make it possible to increase the dose applied to tumours, resulting in an improvement in the cure rate. Along this line, proton minibeam radiation therapy (pMBRT) is a novel strategy that allows the spatial modulation of the dose, leading to minimal damage to brain structures compared to a high dose (25 Gy in one fraction) of standard proton therapy (PT). The aim of the present study was to evaluate whether pMBRT also preserves important cerebral functions. Comprehensive longitudinal behavioural studies were performed in irradiated (peak dose of 57 Gy in one fraction) and control rats to evaluate the impact of pMBRT on motor function (motor coordination, muscular tonus, and locomotor activity), emotional function (anxiety, fear, motivation, and impulsivity), and cognitive function (learning, memory, temporal processing, and decision making). The evaluations, which were conducted over a period of 10 months, showed no significant motor or emotional dysfunction in pMBRT-irradiated rats compared with control animals. Concerning cognitive functions, similar performance was observed between the groups, although some slight learning delays might be present in some of the tests in the long term after irradiation. This study shows the minimal impact of pMBRT on the normal brain at the functional level.

## Introduction

Radiotherapy (RT) is one of the main choices for cancer treatment^1^. The remarkable achievements in dose conformation in the last decades^2^ resulted in an improvement of the therapeutic index of RT treatments. However, the treatment of radioresistant tumours is not yet satisfactory.

The reduced risk of normal tissue complications observed for spatially fractionated radiotherapy techniques^3–8^ could be exploited to find an effective therapeutic strategy for these challenging cases. Along this line, a novel approach called proton minibeam radiation therapy (pMBRT) has been proposed^9^. In contrast to standard proton therapy, irradiation in pMBRT is performed with narrow beams (diameter ≤ 1 mm) separated by gaps of 2 to 4 mm^9^. This results in a lateral dose profile consisting of a series of high doses (peaks) and low doses (valleys). The use of protons instead of photons leads to a negligible dose being deposited in normal tissues after the Bragg peak (tumour position), further reducing the secondary effects. Moreover, recent studies have showcased the distinct biological properties of protons^10^.

The reduced toxicity of pMBRT compared to standard PT might improve the therapeutic index by reducing side effects in cases of paediatric astrocytoma and meningioma with a good rate of tumour control but substantial side effects, such as reduced speech, motor function or intelligence quotient. In addition, it could open the possibility for more efficient treatment of radio-resistant tumours, such as high-grade gliomas, which is still one of the most challenging types of tumours in clinical oncology. Indeed, at the dose used in the present evaluation (25 Gy in one fraction), pMBRT has been shown to lead to equivalent or superior tumour control to standard PT^11,12^ in experiments on glioma-bearing rats.

A recent study compared the responses of two groups of normal rats that underwent irradiation of the whole brain (except the olfactory bulb) at a high dose with either seamless proton irradiation (standard, mean dose of 25 Gy in one fraction) or pMBRT irradiation (peak dose of 57 Gy in one fraction, which corresponds to an average dose of 25 Gy). After a follow-up of 6 months, severe skin and brain damage was observed in the rats treated with conventional proton therapy, while the animals that underwent pMBRT exhibited less severe lesions^3^.

Whether this absence of morphological damage is accompanied by the preservation of brain functions should be determined before clinical trials are conducted. The aim of the present work was thus to perform the first comprehensive behavioural study to assess the impact of pMBRT on motor, emotional, and cognitive functions.

## Materials and methods

### Animals

This study was performed using twenty 6-week-old male Fisher rats (Janvier Labs), which were followed up for a period of 12 months. The animals were irradiated at the Orsay Proton Therapy Centre and then transferred to the animal facility at Institut de Biologie Animale Intégrative et Cellulaire (IBAIC), where they were acclimatized for 2 weeks before the first behavioural tests. The rats were housed in groups of three per cage in a temperature- and humidity-controlled colony room and maintained on a 12:12-h light/dark cycle with ad libitum access to water and food.

All behavioural tests were performed during the day by the same experimenter. The experiments were performed at approximately the same hour each day for each animal to avoid disrupting the sleep cycle. The tests were conducted at least 24 h apart.

### Irradiation

Irradiation was performed with a 100-MeV proton beam. The 20 rats were divided randomly into 2 groups (n = 10), namely, the pMBRT group and the control (non-irradiated) group. The animals of the pMBRT group were anaesthetised (isoflurane, 2.5% in air) for 45 min and received the same dose as in our previous study^3^ (57 ± 3 peak dose and 8.8 ± 0.4 Gy valley doses at a depth of 1 cm, which corresponds to an average dose of 25 ± 1 Gy in one fraction), which led to significant tumour control in glioma-bearing rats^12^. The whole brain, except the olfactory bulb, was irradiated with five 2 cm-high planar minibeams. The minibeams’ width at a depth of 1.0 cm (centre of the rat brain) was 1,100 ± 50 μm.

The control group was anaesthetized for 45 min to achieve the same conditions as in the irradiated group.

We chose to not re-evaluate the effect of standard seamless proton irradiation, since severe brain damage was observed in our previous work at this dose^3^.

### Experimental procedures and morphological measurements

**Figure 1:**
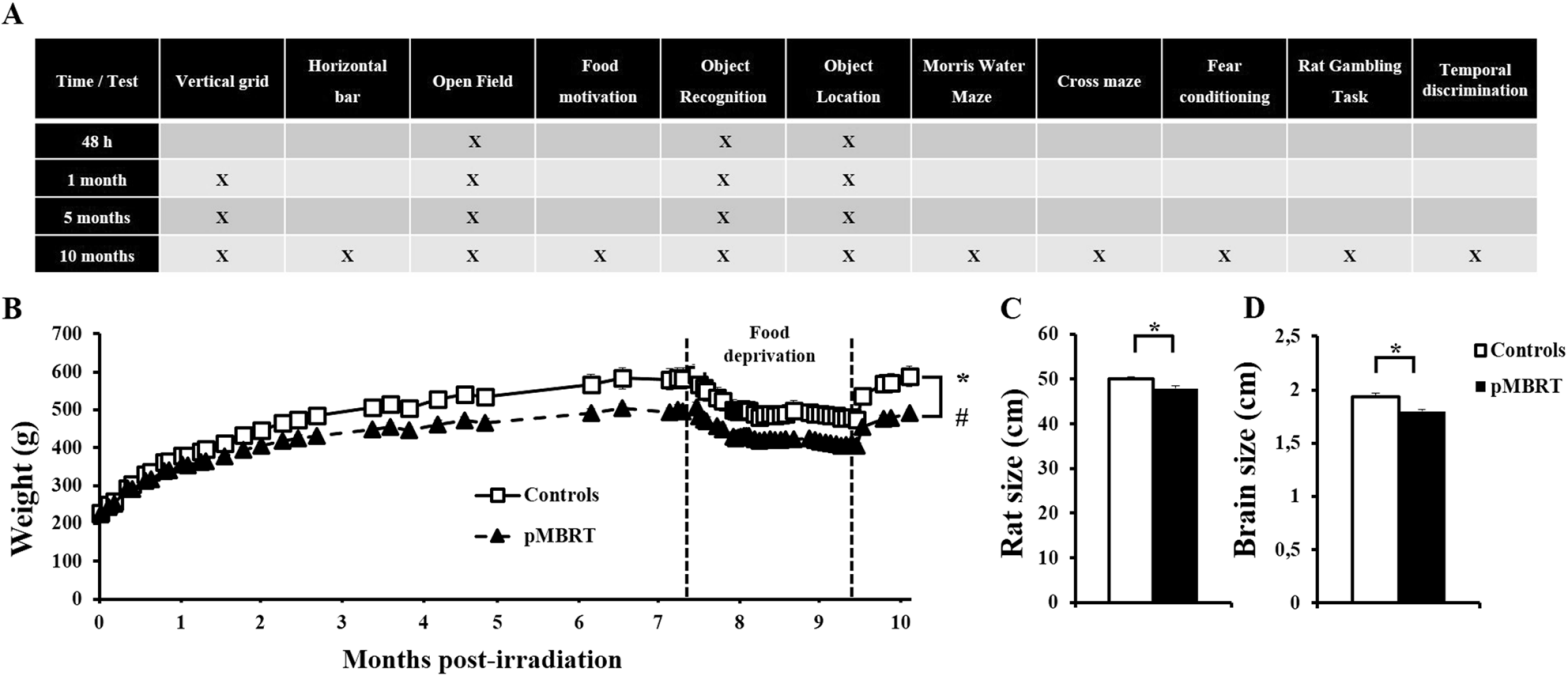
Morphological measurements. General overview of the behavioural tasks (**A**). Irradiated rats were lighter than control rats as a function of days post-irradiation (PI) (**B**). pMBRT rats had smaller bodies (**C**) and brains (**D**) than control animals at 10 months PI. The data are presented as the mean ± SEM; *p < 0.05; #: group × month interaction.

Figure 1A shows a general overview of the behavioural tests performed. This longitudinal study assessed the behaviour of the 20 rats in the short term (1 month) until the long term (10 months) after irradiation. Some tests could be repeated several times, whereas others [the Morris water maze (MWM) test, cross-maze test, rat gambling task (RGT), temporal discrimination test, and fear conditioning test] could not due to learning biases. These tests were performed only once at 10 months post irradiation (PI) to evaluate the long-term effect of pMBRT. A detailed description of the protocols of all the performed tests can be found in the Supplemental Material.

The weight of each rat was monitored throughout the study. At the end of the study (11 months PI), the size of each rat (body and tail) was measured. Then, the rats were intracardially perfused and the length of each freshly dissected brain was also measured.

### Behavioural tasks

A brief description of all the tests performed is provided hereafter. A more detailed explanation of the methodology can be found in Supplemental Material.

#### Basal assessment: motor ability, anxiety, and motivation

Motor coordination was assessed based on the latency to body reversal (“return”) in the vertical grid test. Muscular tonus was evaluated using the horizontal bar test, in which the latency for each rat to fall from the bar was recorded.

The open field (OF) test was adopted to measure locomotor activity and anxiety. Each rat was placed in an open arena and allowed to freely explore. Anxiety was inversely correlated with the time the rat spent in the centre of the arena. Each animal was placed in the OF arena three times per day (for 5, 5, and 3 min) with an interval of 3 h between each trial. The total distance travelled, and the time spent in the centre were recorded.

Motivation for food was also assessed. Each rat was placed individually in a closed arena containing two small cups with 5 g of either normal or chocolate-flavoured pellets. The quantity of pellets consumed in 5 min was assessed. This test was performed twice: when rats were under food deprivation (maintained at 85% of their initial weight), and 1 week later, when rats were fed ad libitum.

#### Memory assessment

The object recognition task (ORT) and object location task (OLT) were performed to evaluate the ability of the rats to recognize a novel object or location in a known environment. Each rat was allowed to familiarize itself with two identical objects in the OF arena twice (3-h interval) for 5 min. Three hours later, the rat was placed in the OF arena for 3 min with one novel object (ORT) or with the same object moved to a new location (OLT) and the same familiar object in the same location. The time spent exploring each object and the total distance travelled were measured and used to calculate the discrimination ratio ([time exploring the novel object or the novel location of the object—time spent exploring the familiar object or location]/time spent exploring both objects).

The Morris water maze (MWM) task was used to investigate spatial learning and memory using a standard protocol^13^. The training phase, in which each rat was required to find a platform, lasted 9 days (4 trials/day, 36 trials in total). The distance travelled to find the hidden platform was recorded. On the tenth day, the platform was removed, and a 60-s probe trial was performed. The distance travelled in each quadrant was recorded.

The cross-maze test^14^ was used to assess the contributions of response (egocentric) and place (allocentric) learning strategies to memory. A T-maze (with east [baited], west, and south arms) was created. Each rat started from the south arm and had to learn to travel to the east arm to obtain a reward, i.e. food pellets. Each rat underwent four trials per day, and the number of correct responses (east arm) was measured. When an animal achieved the criterion (six correct choices on two consecutive days), a probe test was performed the next day. In the probe test, the south arm was closed, and the north arm was used as the new starting arm. The strategy, place (spatial response, east arm) or response (turn-right response, west arm) that the animal used to reach the goal arm was assessed.

A Pavlovian fear conditioning task^15^ was used to study potential alterations in associative learning or emotional reactivity. On the first day, each animal was placed in an operant box in which a tone (CS) followed by a short electrical foot shock was given. The session consisted of five tone-shock pairings. During the next 2 days, each rat was placed in a new context (modified box) in which 16 CSs alone (extinction) were delivered. Each day, a camera was used to record the behaviour of the rats, and the freezing rates in response to the CSs were scored off-line.

#### Executive functions assessment

Decision making and impulsivity were evaluated using the Rat Gambling Task (RGT)^16,17^. In this test, each rat was given four options (holes to poke with its nose) reinforced by an immediate reward. Two options were associated with large reward (2 food pellets) but were disadvantageous in the long term due to large penalties (time out). Two other options were associated with smaller reward (1 food pellet), but smaller penalties, and were thus more advantageous over the entire session. These contingencies were arranged to create a conflicting situation between the reward size during each trial and the overall quantity of the reward over the session. Based on the last period of the test, each rat was categorized as: (1) low-impulsive (LI) if it made at > 70% of the advantageous choices; or (2) high-impulsive (HI) if it made at < 25% of the advantageous choices. The other animals, performing between 25 and 70% were not categorized.

In the temporal discrimination task^18^, the rats were trained to press a lever on the left or right side after the presentation of a 2- or 8-s tone, respectively. Then, a psychophysical choice procedure (bisection test), in which five intermediate durations (2.5, 3.2, 4, 5, and 6.3 s) were presented as well as the two trained anchor durations (2 and 8 s), was conducted. Correct responses to the anchor durations were reinforced, whereas errors and responses to the intermediate durations were not.

### Histopathological assessment

At the end of this study, each rat was anaesthetized, and blood was collected. After intracardiac perfusion, the brain was extracted, fixed in 10% neutral buffered formalin, and embedded in paraffin. Then, 4 μm-thick parasagittal sections of four (irradiated rats) or two (control rats) different regions of the left and right parts of the brain of each rat were cut and stained/labelled. The analyses performed on sections from each region are described in Table 1. We focused describing the histological lesions (hematoxylin and eosin (HE) staining) and evaluating (i) microglial cells (immunohistochemistry (IHC) for ionized calcium-binding adapter molecule 1 immunohistochemistry (Iba-1) using an anti-rabbit Iba-1 antibody; Wako, #019-19741, 1:1,000), (ii) astrocytes (IHC for glial fibrillary acidic protein (GFAP) using an anti-chicken GFAP antibody; Abcam, #ab4674, 1:5,000), (iii) apoptosis (IHC for activated caspase 3 using an anti-rabbit activated caspase-3 antibody; Cell Signaling, #9664, 1:200), and (iv) myelin (luxol fast blue staining).

**Table 1:** Histopathological assessment.

Levels of section	1	2	3	4
Control (left and right brain)	H&E, IBA-1, GFAP, Act. Casp. 3, LFB	/	/	H&E, IBA-1, Act. Casp. 3
pMBRT (left and right brain)	H&E, IBA-1, GFAP, Act. Casp. 3, LFB	H&E, IBA-1	H&E, IBA-1	H&E, IBA-1, Act. Casp. 3

Histopathological analyses were performed in each section from each region for the control (2 regions separated by at least 600 µm) and irradiated (4 regions separated by at least 200 µm) groups. HE (haematoxylin and eosin) staining to evaluate the lesions. Iba-1 (ionized calcium-binding adapter molecule 1; marker of microglial cells), GFAP (glial fibrillary acidic protein; marker of astrocytes), Act. Casp. 3 (activated caspase 3; marker of apoptosis), and LFB (luxol fast blue; myelin) staining.

Microglia activation was quantified using automated detection of the Iba-1 signal and by measuring the labelled area on one entire brain section per rat using ImageJ software (https://imagej.nih.gov/ij/). Reactive microglial cells display a larger cell body and thicker cell processes (or small or no cell processes; ameboid form); they can also be grouped as clusters (microglial nodules) in tissue. These modifications generally increase (at least focally) the Iba-1 immunohistochemical signal.

### Cytokine expression

The blood levels of pro-inflammatory cytokines (IL1β and IL6) were quantified by an ELISA kit (Thermo Fisher Scientific) according to the provider’s protocol with pure serum.

### Statistical analysis

Statistical analyses were performed using the software JASP^19^ with a threshold of 0.05. Fisher’s exact test and Student’s t test were also used. Bayesian ANOVAs and independent sample t-tests were further conducted to characterize the findings.

### Ethics statement

All animal experiments were conducted in accordance with the animal welfare and ethical guidelines of our institutions. They were approved by the French Ministry of Research (permit no. 16750–2018091215388677 v2).

## Results

### Morphological measurement

Figure 1B shows the evolution of the weight of the rats as a function of days after treatment (PI). All the animals showed an increase in weight (controls: F(30,270) = 192.05, p < 0.001; pMBRT: F(30,270) = 608.17, p < 0.001), however, the irradiated rats were 12.2% lighter than the control rats (F(1,18) = 6.39, p < 0.05), and a significant group x day interaction was observed (F(30,540) = 9.80, p < 0.001). A Bayesian repeated measures ANOVA revealed that the group x day interaction model was preferred to the main effects model by a Bayes factor of 1.602e + 31, confirming the differential growth between groups. Weights were significantly different between groups from the 39th day PI (F(1,18) = 4.55, p < 0.05). The pMBRT rats had smaller bodies (4.2%) and brains (6.9%) than the control animals (Fig. 1C: t(17) = 3.14, p = 0.003; Fig. 1D: t(17) = 3.33, p = 0.002). Bayesian independent samples T-test further confirmed these results (Fig. 1C: BF_10_ = 8.284; Fig. 1D: BF_10_ = 12.60) with respectively moderate and strong evidence for the alternative hypothesis. These results suggest growth retardation of the irradiated animals.

### Behavioural tasks

#### Basal assessment: motor ability, anxiety, and motivation

**Figure 2:**
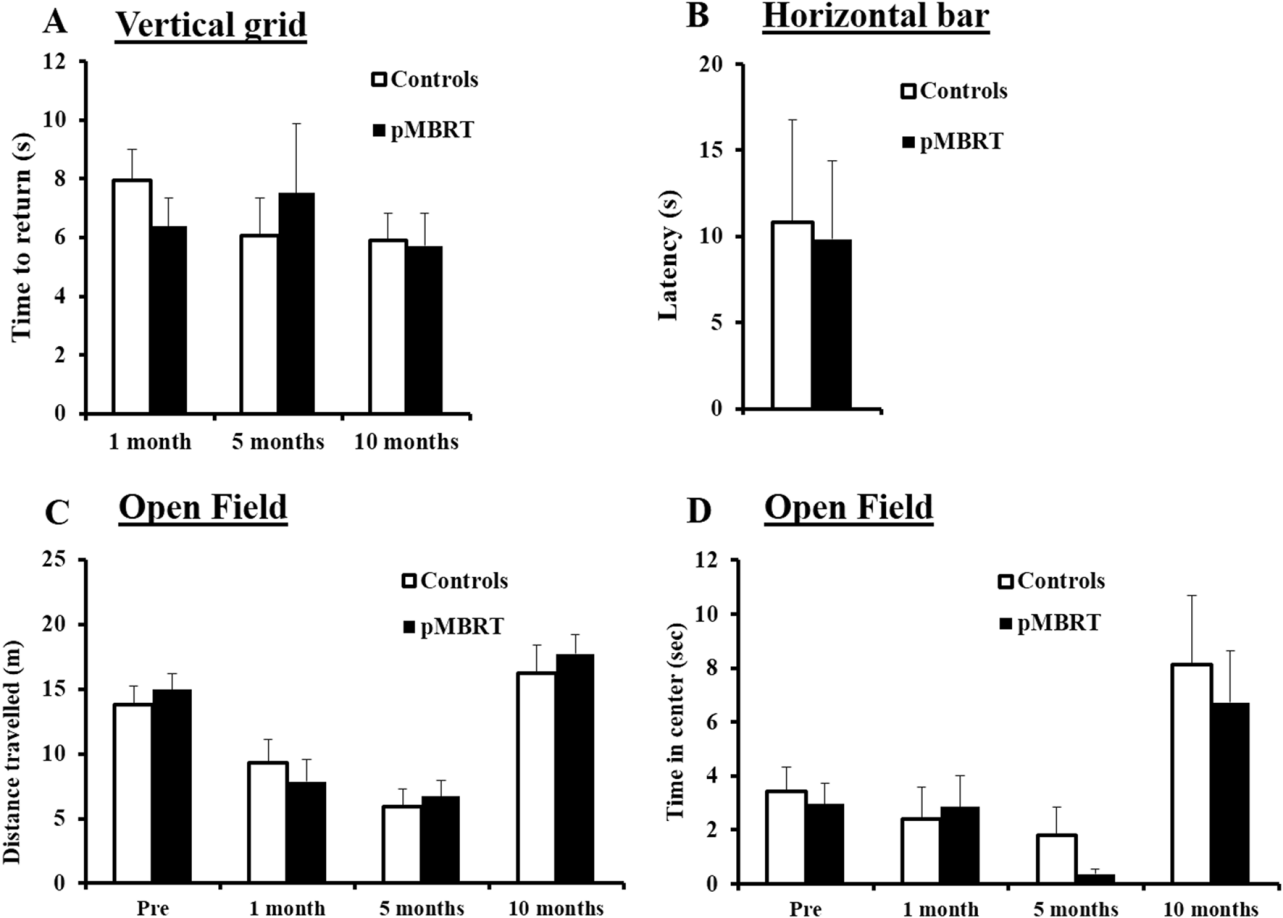
Motor and anxiety assessments. Similar mean time required to return on the vertical grid between the groups at 1, 5, and 10 months PI (**A**). Similar mean latency to fall from the horizontal bar between the groups at 10 months PI (**B**). Similar mean distance travelled (**C**) and mean time spent in the centre zone (**D**) between the groups in the OF test at 48 h before irradiation and 1, 5, and 10 months PI. The data are presented as the mean ± SEM.

For the motor coordination (Fig. 2A), a 2 (group) × 3 (month) ANOVA on the time required to return on the vertical grid by the 2 groups (control and pMBRT rats) as a function of months PI (1, 5 and 10 months) yielded no main effect of group, no main effect of month and no group × month interaction (Fs < 1). The Bayesian repeated measures ANOVA was also in favor of the null hypothesis (all 0.029 < BF_10_ < 0.363). These results show that the irradiation did not affect motor coordination.

Similarly, muscular tonus was not impacted by irradiation (Fig. 2B); no difference in latency to fall between the groups in the horizontal bar test (t(18) = 0.13, p = 0.45) was observed. This was confirmed by the Bayesian independent samples T-test (BF_10_ = 0.416) in favor to the null hypothesis.

**Figure 3:**
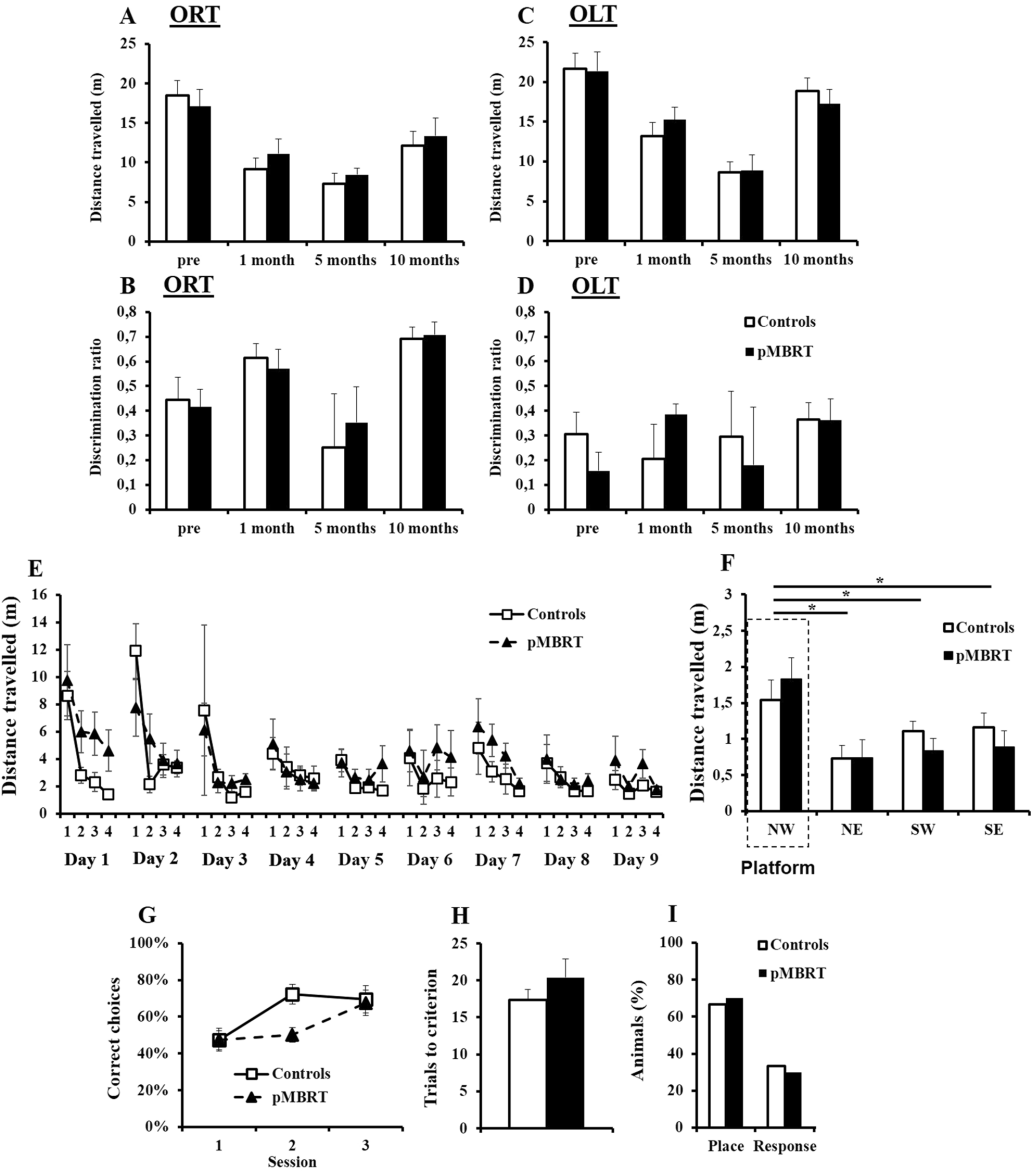
Memory assessment. Similar mean distance travelled by both groups in the ORT (**A**) and OLT (**C**) at 48 h before irradiation and 1, 5, and 10 months post-irradiation (PI). Similar mean discrimination ratio for both groups in the ORT (**B** (time spent exploring the novel object – time spent exploring the familiar object)/time spent exploring both objects) and in the OLT (**D** (time spent exploring the novel location of the object – time spent exploring the familiar location)/time spent exploring both objects) at 48 h before irradiation and 1, 5, and 10 months PI. Similar mean distance travelled to find the platform across trials by both groups in the MWM test at 7 months PI (**E**). Similar mean distance travelled in each quadrant of the MWM by both groups during the probe test (**F**). Similar mean percentage of correct choices to find the east arm (**G**) and mean number of trials needed to reach the learning criterion (**H**) during the training sessions of the cross-maze test for both groups. The majority of animals showed a place strategy (east arm) rather than a response strategy (west arm) during the probe test, and there was no significant difference between the groups (**I**). The data are presented as the mean ± SEM; *p < 0.05.

Locomotor activity was also not altered as measured by the OF test, ORT, and OLT (Figs. 2C, 3A,C). Indeed, analysis of distance travelled yielded no main effect of group, no main effect of month and no group × month interaction (Fs < 1). This was confirmed by Bayesian repeated measures ANOVA as Bayes factors for the factor group were in favor of the null hypothesis (OF: BF_10_ = 0.296; ORT: BF_10_ = 0.324; OLT: BF_10_ = 0.308). Incidentally, locomotor activity decreased as the tests were repeated and then increased at 10 months PI for both groups. Indeed, in a relatively short time (5 months), the animals were exposed to the same OF apparatus three times, so habituation to this environment led to decreased exploratory activity. The increased locomotor activity at the 10-month time point can be explained by the large number of tests conducted at this point, which led to increased global activity^20^.

Analysis of the time spent in the centre of the OF arena (Fig. 2D) indicated no difference in anxiety levels between the two groups as a 2 (group) × 3 (month) ANOVA yielded no main effect of group, no main effect of month and no group x month interaction (Fs < 1). Bayesian repeated measures ANOVA confirmed the lack of effect as the Bayes factor for the factor group (BF_10_ = 0.328) was in favor of the null hypothesis.

Concerning food consumption (data not shown), a 2 (group) × 2 (flavour) ANOVA on quantity of food pellets consumed by both groups (control and pMBRT rats) as a function of the flavour of pellets (normal or chocolate) yielded no main effect of group, no main effect of flavour (Fs < 1) and no group x flavour interaction (food deprivation: F(1,17) = 2.36, p = 0.14; ad libitum regimen: F(1,17) = 0.18, p = 0.68). These results were confirmed by Bayesian repeated measures ANOVA, since Bayes factors for the factor group (food deprivation: BF_10_ = 0.365; ad libitum regimen: BF_10_ = 0.359) were in favor of the null hypothesis. These results show that the irradiation had no effect on motivation for food under both food regimen (deprivation and ad libitum).

#### Memory assessment

Object recognition and spatial memory were unaltered in the irradiated animals (Fig. 3A–D). All the animals exhibited a positive discrimination ratio (one sample T-test: t_s_ > 2.35, p_s_ < 0.05), suggesting good memory of the familiar object and location. No group difference or group × month interaction was revealed (Fs < 1). This was confirmed by the Bayesian repeated measures ANOVA with Bayes factors for the factor group in favor to the null hypothesis (ORT: BF_10_ = 0.303 and OLT: BF_10_ = 0.311).

Regarding spatial learning and memory in the MWM test, neither a significant group effect (F(1,18) = 2.25, p = 0.15), nor a group × day interaction (F(8,144) = 1.17, p = 0.32) was found during the training phase (Fig. 3E). Bayesian repeated measures ANOVA confirmed the lack of group difference as the Bayes factor (BF_10_ = 0.372) was in favor of the null hypothesis. The distance travelled to find the platform decreased as the number of training days increased (F(8,144) = 6.6, p < 0.001), suggesting that all animals learned the platform location. However, an in-depth analysis of the first training day indicated that the irradiated rats travelled a longer distance than the control animals to find the platform (F(1,18) = 6.22; p < 0.05). The group × trial interaction was not significant (F < 1), but the distance travelled by the control animals (F(3,27) = 11.85, p < 0.001) but not by the pMBRT rats (F(3,27) = 1.77, p = 0.18) decreased across the four trials. These results suggested that irradiation induced some spatial learning delay, but limited to the first session. In the probe test (Fig. 3F), compared with the control animals, the irradiated animals travelled a similar distance, and no group × quadrant interaction was found (Fs < 1). Bayesian repeated measures ANOVA confirmed the lack of group difference as the Bayes factor for the factor group (BF_10_ = 0.314) was in favor to the null hypothesis. Furthermore, the distance travelled in the target quadrant (NW) was increased compared with that in the other quadrants (NW vs. NE: F(1,18) = 14.31, p < 0.01 ; NW vs. SW: F(1,18) = 10.10, p < 0.01 ; NW vs. SE: F(1,18) = 6.89, p < 0.05). Therefore, the spatial memory of both the control and pMBRT rats was maintained.

Concerning spatial strategy in the cross-maze test, the number of correct choices increased with training for both groups (Fig. 3G: control rats: F(2,16) = 8.23, p < 0.01; pMBRT rats: F(2,18) = 4.5, p < 0.05). Although there was no group effect (F(1,17) = 2.12; p = 0.16), the trend of the group × session interaction (F(2,34) = 3.10, p = 0.058) suggested that the pMBRT rats exhibited a slight learning delay. This result was confirmed by Bayesian repeated measures ANOVA as the group x session interaction model was preferred to the main effects model by a Bayes factor of 1.741. Nevertheless, the number of trials needed to reach the criterion did not differ between the groups (Fig. 3H: t(17) = 1.12, p = 0.14), this result was confirmed by a Bayes factor (BF_10_ = 0.615) was not in favor of the alternative hypothesis.

During the probe test (Fig. 3I), most of the animals (67% of controls and 70% of pMBRT rats) adopted a place strategy, while the others adopted a response strategy; there was no significant difference between the groups (Fisher exact test: F = 1, p > 0.05), suggesting that irradiation did not influence the chosen strategy.

**Figure 4:**
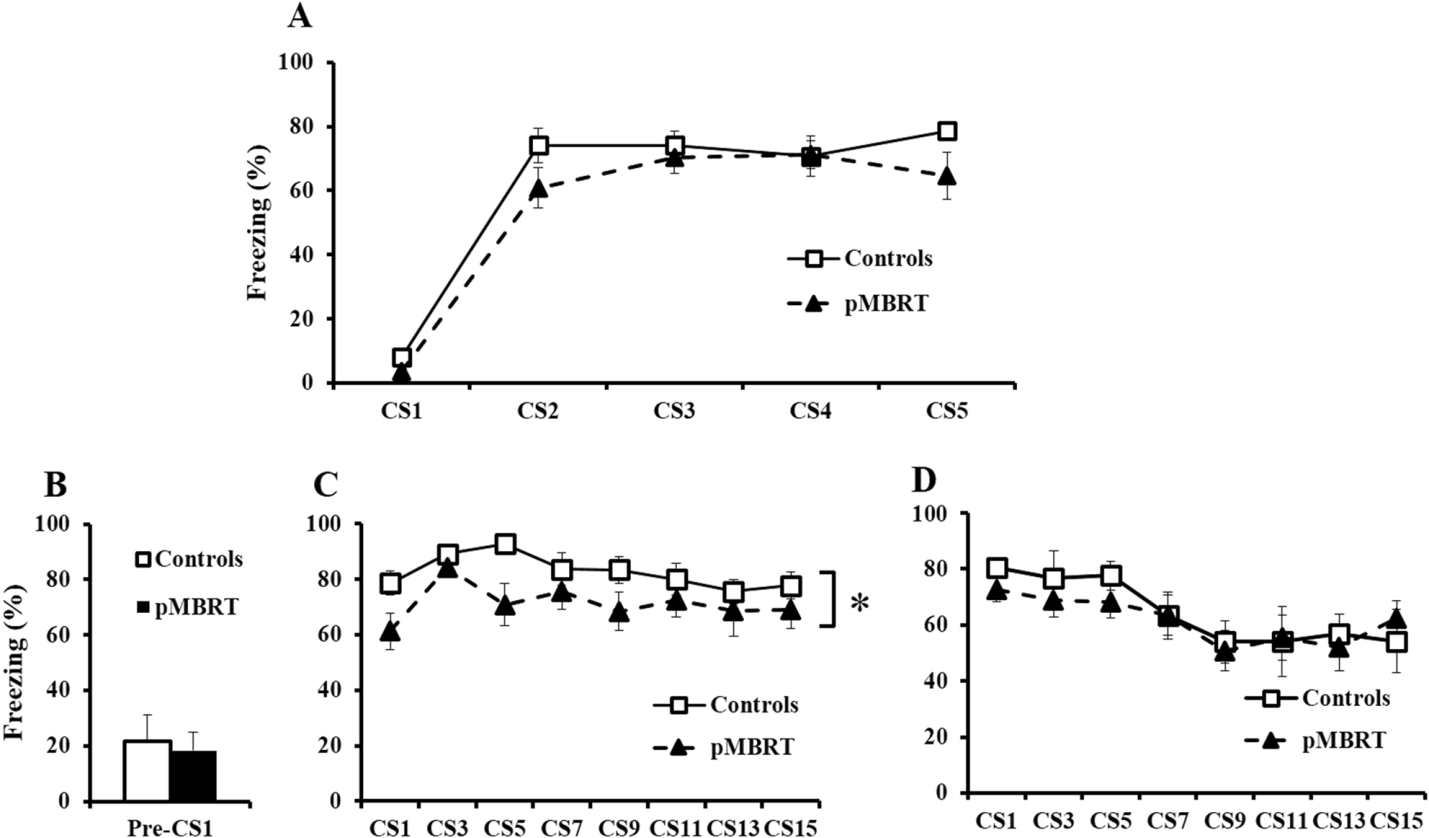
Fear conditioning test. Similar mean percentage of freezing during CS presentation on the conditioning day (**A**) and during the 30 s preceding the first CS presentation of the first extinction day in another context (**B**) for both groups. pMBRT rats had a lower mean percentage of freezing than control rats during CS presentation on the first extinction day (**C**), but not on the second extinction day (**D**). The data are presented as the mean ± SEM; *p < 0.05.

In the fear conditioning task, the freezing rate during the first tone presentation (CS1) of the conditioning day (context A, Fig. 4A) reflected basal reactivity to a neutral stimulus. The freezing level during the following CS presentations indicated associative learning. Irradiation did not affect freezing (F(1,17) = 3.0, p = 0.10), and no group × CS interaction was observed (F(4,68) = 1.19, p = 0.32), but a significant effect of repeated CSs (F(4,68) = 98.94, p < 0.001) suggested good and similar associative learning for both groups. Bayesian repeated measures ANOVA confirmed the lack of a group difference with a Bayes factor (BF_10_ = 0.382) not in favor of the alternative hypothesis. The percentage of freezing during the 30 s preceding the first CS presentation (Pre-CS1, Fig. 4B) reflected the basal anxiety level of the animals and/or fear generalization in the new context B, and no group difference was identified (t(17) = 0.31, p = 0.38) as confirmed by the Bayesian independent samples T-test (BF_10_ = 0.419) not in favor of the alternative hypothesis. When the CS was repeatedly presented in the extinction phase, a global group effect was evident on the first day of extinction (Fig. 4C: F(1,17) = 5.45, p < 0.05) but not on the second day (Fig. 4D: F < 1) as confirmed by the Bayesian repeated measures ANOVA with a group factor in favor of the alternative hypothesis for the first day in extinction (Fig. 4C: BF_10_ = 1.983) and in favor of the null hypothesis for the second day (Fig. 4D: BF_10_ = 0.334). The effect of the CS on both days (Fig. 4C: F(7,119) = 2.87, p < 0.01; Fig. 4D: F(7,119) = 4.69, p < 0.001), for which there was no group x CS interaction (Fs < 1), suggested a similar rate of extinction between the groups across the two sessions. Bayesian repeated measures ANOVA confirmed that the main effects model was preferred to the group x CS repetition interaction model by a Bayes factor of 7.41 for the first day of extinction and 13.70 for the second day.

#### Executive functions assessment

**Figure 5:**
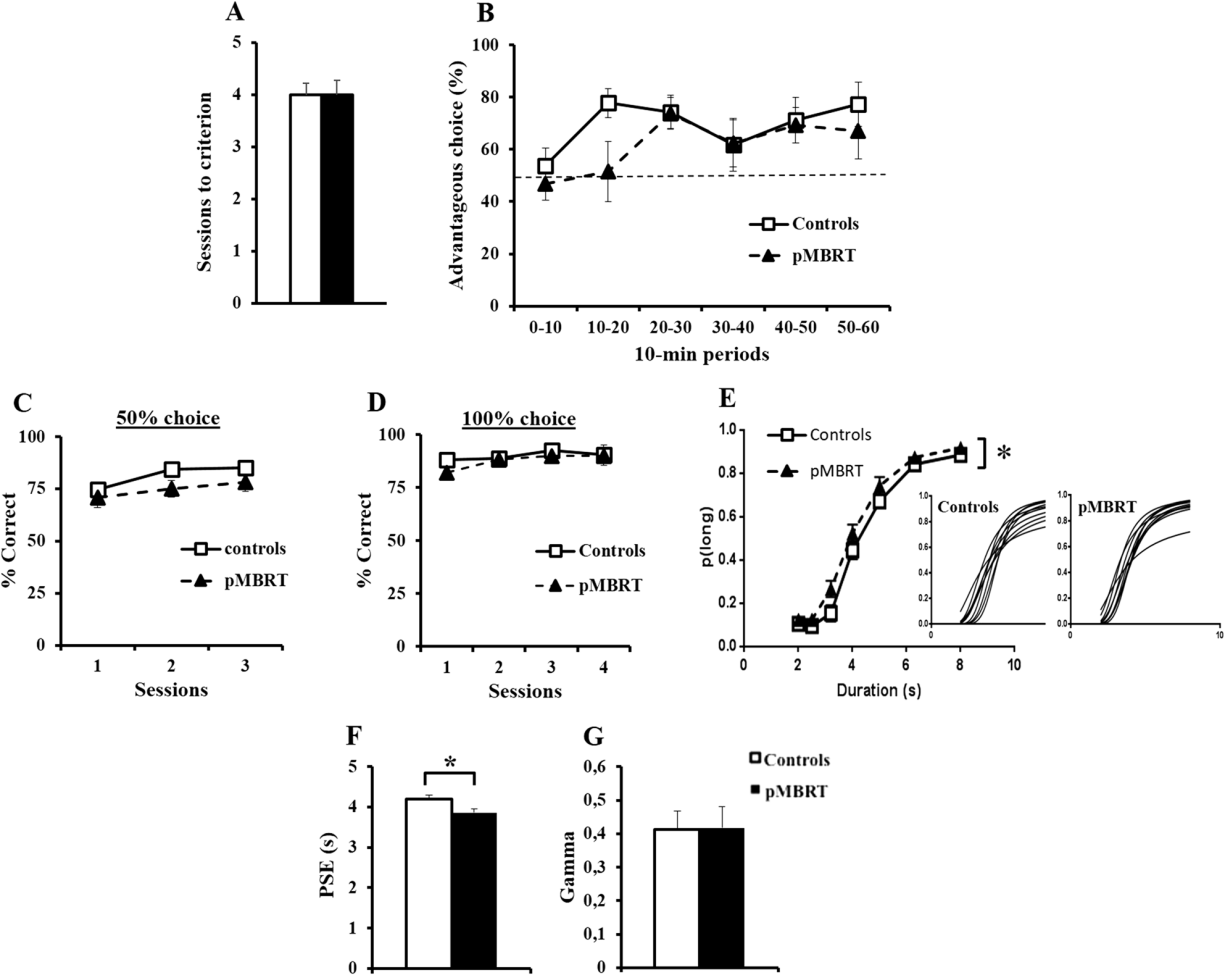
Executive functions assessment. Similar mean number of sessions needed to reach the criterion during RGT training for both groups (**A**). The mean percentage of advantageous choices in 10-min periods during the RGT session (dotted line: random choice) was not significantly different between the groups (**B**). Similar mean percentage of correct responses for both groups during the training sessions of the temporal discrimination task with 50% (**C**) and 100% (**D**) free choice. Compared to control animals, pMBRT animals showed a higher mean proportion of long responses (p(long)) as a function of stimulus duration during the bisection tests (insets: individual curves) (**E**). The point of subjective equality (PSE) was lower in pMBRT animals than in control animals (**F**), while the temporal precision (gamma) was not significantly different (**G**). The data are presented as the mean ± SEM.

During the training phase of the RGT (Fig. 5A), the control and pMBRT rats required the same number of sessions to achieve the learning criterion (t(1,18) < 0.001, p = 0.5). This result was confirmed by the Bayesian independent samples T-test (BF_10_ = 0.397) in favor of the null hypothesis.

No significant difference in the number of advantageous choices during the RGT session (Fig. 5B) was observed between the groups (F < 1), and there was no group × period interaction (F(5,90) = 1.68, p = 0.15). The Bayesian repeated measures ANOVA confirmed the lack of group difference with a Bayes factor (BF_10_ = 0.314) in favor of the null hypothesis. Moreover, the main effects model was preferred to the group x period interaction model by a Bayes factor of 1.86. The rate of advantageous choices made by the rats over the six 10-min periods increased (F(5,90) = 5.11, p < 0.001), suggesting that the rats had a good understanding of the task and that both groups developed an efficient strategy. A second ANOVA analysis without the first 10-min period values was also performed. This new analysis showed no main effect of group (F(1,18) = 0.70; p = 0.42) and no group × period interaction (F(4,72) = 2.2; p = 0.076), confirming the first analysis including all periods.

The percentage of low-impulsive rats was not significantly different between the groups (data no shown, Fisher exact test: F = 1, p > 0.05), and no rat was categorized as high-impulsive (advantageous choice < 25%).

Concerning the temporal discrimination task, there was neither a group effect (Fig. 5C: F(1,18) = 2.49, p = 0.13; Fig. 5D: F(1,18) = 1.21, p = 0.29) nor a group x session interaction (Fs < 1) during the training sessions. Bayesian repeated measures ANOVAs confirmed the lack of group difference as the Bayes factors (Fig. 5C: BF_10_ = 0.986 and Fig. 5D: BF_10_ = 0.429) were not in favor of the alternative hypothesis. The increasing (Fig. 5C: F(2,36) = 9.29, p < 0.001) and high percentage of correct responses during training (Fig. 5C,D) suggested good and similar learning for both groups.

With respect to the bisection test session (Fig. 5E), based on the proportion of responses given to the correct lever for the long-duration stimulus, the temporal performance of the pMBRT animals differed from that of the controls; the pMBRT rats had a higher p(long) than the controls (F(1,18) = 8.43, p < 0.01), but no group × duration interaction was found (F < 1). However, a Bayesian repeated measures ANOVA suggests a lack of group difference as the Bayes factor for group (BF_10_ = 0.287) was in favor of the null hypothesis. The point of subjective equality (PSE, Fig. 5F) was lower in the pMBRT rats than in the control rats (t(18) = 2.46, p = 0.012) as also suggested by the Bayesian independent samples T-test with a close to moderate evidence in favor of the alternative hypothesis (BF_10_ = 2.750). This result confirm that irradiation affected bisection performance. With regard to temporal precision (gamma, Fig. 5G), no significant effect of irradiation was found (t(18) = 0.05, p = 0.48). This result was confirmed by the Bayesian independent samples T-test (BF_10_ = 0.398) not in favor of the alternative hypothesis. That meaning that all rats showed equal temporal precision.

### Histopathological assessment

**Figure 6:**
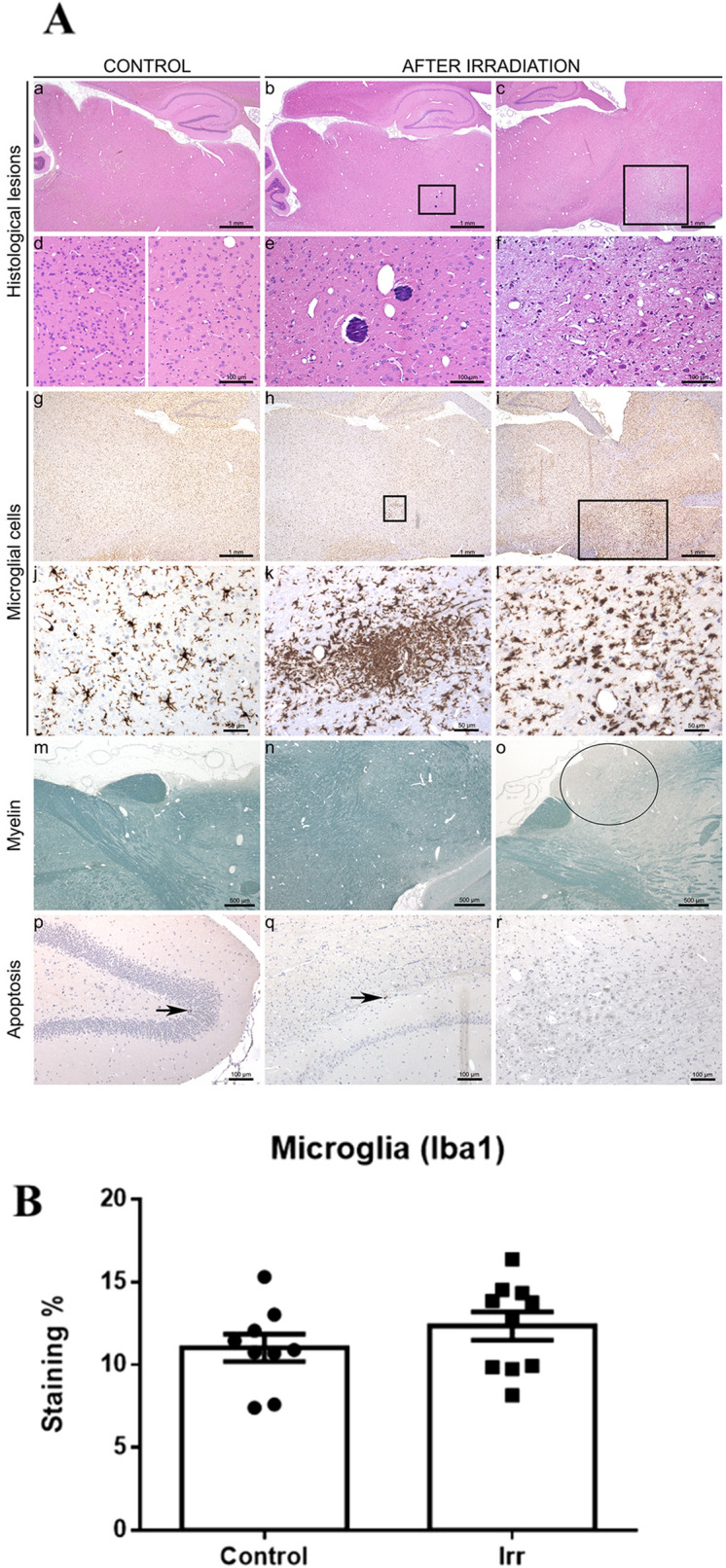
Histopathological assessment. At low magnification, no lesions were observed in the brains of control rats (**Aa**). After pMBRT, multifocal lesions were detected in random locations in some rats. The exact localization of the lesions in para-sagittal sections was hard to define; some lesions were detected in the thalamus (**Ab**) or extending from the olfactory bulb/structures (**Ac**). These lesions were characterized by spongiosis, gliosis, foci of mineralization (thalamus high magnification, **Ae**) and less frequently neuropil necrosis (extension of olfactory bulb/structures high magnification, **Af**). Note the normal aspect of brain tissue in the extension of olfactory bulb/structures (left image) and the thalamus (right image) at a high magnification (**Ad**). Microglia morphology analysed by immunohistochemistry for Iba-1 revealed resting microglial cells with thin and long cell processes for control rats (**Ag,j**) and microglia reaction and microglial nodule formation (**Ah,i,k,l**), with shorter and thicker cell processes and hyperplasia, after pMBRT. Concerning myelin, a focus of myelin pallor, which indicated alteration of the myelin structure, was detected in the cortex (**Ao**) of one rat after pMBRT. Normal myelin staining (**Am**,**n**). Rare apoptotic neurons were detected in control and irradiated rats (black arrows, **Ap**,**q**,**r**). The quantification of the averaged Iba-1 signal (linked to microglial activation) revealed no significant difference between the groups (**B**).

Histopathological analysis of the brain revealed few differences between the groups (Fig. 6A). In the pMBRT group, we observed (i) small calcifications of the neuropil (7/10 rats, approximately 100 µm in diameter), (ii) edema/spongiosis (7/10 rats), (iii) scattered nodules of reactive microglial cells (observed in both groups but larger after pMBRT, especially in 7/10 rats), and (iv) small necrotic foci, sometimes with neutrophils (4/10 rats). Most of the lesions were very small (~ 100 µm, well delimited), with only one rat exhibiting a larger lesion (Fig. 6Ac,f,i,l). The quantity of apoptotic neurons, which were detected using immunohistochemistry for activated caspase-3, was similar between the two groups.

**Figure 7:**
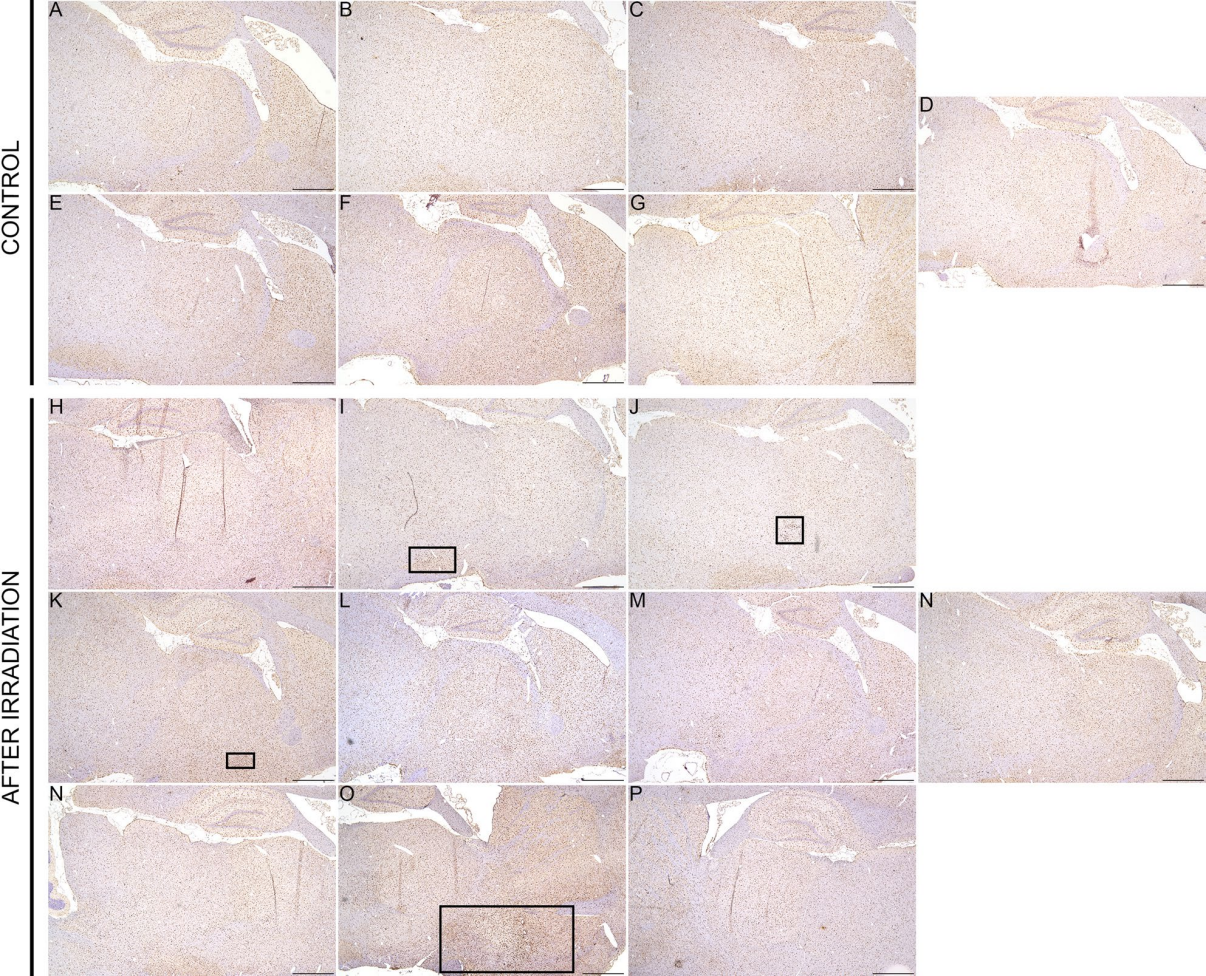
Low magnification (Iba1 immunohistochemistry) of sagittal brain sections (scale bar: 1 mm) of each rat showing small loci of reactive microglial cells in three animals (I, J, K), after irradiation (black squares). Only one rat (P) displayed more extensive/severe lesions.

The quantification of the Iba-1 signal revealed no significant differences between the groups (see Fig. 6B), and thus confirming the absence of extensive damage, as it can also be seen in the Fig. 7 (Supplemental materials). Lesions in the pMBRT group were minimal to moderate; no marked inflammation or large areas of necrosis with cavitation were detected.

### Cytokine expression

No measurable levels of IL1β and IL6 were found in the blood serum of either group.

## Discussion

There is extensive clinical evidence of neurocognitive decline in patients after whole or partial irradiation of the brain^21–23^. Radiation-induced deficiencies involve many cognitive domains^24–28^, suggesting that normal tissue injury involves diverse neural regions and systems.

In contrast, pMBRT causes generally less severe histological damages in the irradiated brain, even after the delivery of high doses^3^. The goal of our study was to investigate the impact of pMBRT on the brain functions in a rat model, and to correlate the severity of the histological lesions with a possible alteration of cognitive functions. The impact of pMBRT on whole young and healthy brains should indeed be studied to guarantee that pMBRT does not induce side effects even in the long term after irradiation, and that normal tissue is preserved at the structural and functional levels. Whole brain irradiation can cause a vast majority of structural brain damage. The behavioural tests performed in our study were chosen to test the functionality of the main brain structures (the hippocampus, basal ganglia, amygdala, and prefrontal cortex).

The morphological measurement of our study revealed that pMBRT delivered at 6 weeks of age seemed to lead to some growth perturbation (slightly lower (12.5%) body weight and size). This could be relevant for pediatric patients. Further optimisation of the pMBRT protocols (in terms of dose, geometry, irradiated volume) might allow eliminating this already small perturbation. Diminished PI weight loss^29,30^ in rodents has been reported after conventional RT. The pMBRT might exert some impact on the hypothalamus and/or the pituitary gland^29,31,32^.

Concerning the behavioural assessment, our study revealed no significant difference between the control and pMBRT groups in locomotor activity, motor coordination, or muscular tonus at any time after irradiation. This contrasts with the reduced open field activity and sensorimotor deficiencies found at lower doses of conventional RT in a previous study^33^.

No significant difference between the two groups was observed on anxiety level. In contrast, negative behavioural effects, including depression^34,35^ and anxiety^34,36^, have been identified after conventional RT. Moreover, our results indicate that pMBRT treatment did not induce any visible alterations in other emotional functions, such as motivation, impulsivity, and reactivity to threatening stimuli.

Most of our tests aimed to assess cognitive functions, particularly hippocampus-dependent tasks, which conventional irradiation had been shown to impair^37^. Indeed, in these cases, deficiencies in spatial learning^38–49^ and performance on the ORT^41,50–52^ were reported. In contrast, after pMBRT, we did not detect any major deficiency in spatial learning, spatial memory, spatial strategy or recognition memory at any time after irradiation, suggesting that the integrity of the hippocampus was maintained.

The fear conditioning task showed that pMBRT had no impact on associative learning, which contradicts studies on conventional irradiation at low doses^53^. However, the pMBRT animals showed slightly less freezing in response to CSs than the control rats on the first day of extinction, suggesting some alteration in associative long-term memory.

The functional preservation of the brain structures involved in complex cognitive tasks was also assessed. Successful performance of the RGT requires flexibility in planning for considering various outcomes, working memory for processing incoming information and evaluating the risk-reward ratio, and the ability to avoid choosing options that are immediately more rewarding. Our findings showed no significant difference in these executive functions between the groups, both of which exhibited improved performance over time, suggesting the intact functioning of the fronto-striatal brain circuit.

The evaluation of temporal discrimination, another highly demanding cognitive task in which prefrontal-striatal circuits play a critical role^54^, showed that compared with the control rats, the pMBRT animals exhibited a leftward shift of the bisection curve. Such a shift, which is associated with a lower PSE, may reflect a “long” bias in judgement and/or altered storage of temporal reference memory^55^. However, no difference in the gamma measure was detected. Therefore, irradiation did not alter temporal sensitivity.

Finally, even if no statistically significant differences were observed in most of the tests, prudency is recommended before translating this evaluation to clinical trials. Indeed, a slight and temporary delay seems to be present pMBRT rats compared to control at the very start of the learning phase in some tasks in, even if not validated statistically. In particular, it seemed that the distance traveled to find the platform decreased less rapidly in the pMBRT rats than in control animals during the first learning session of the water maze task, that the number of correct choices are lower for the pMBRT group compared to the control animals during the second training session in the cross-maze test, and that the pMBRT animals tended to need more time than did the control rats to reach 70% of advantageous choices in the RGT. These observations might witness very mild cognitive alterations possibly due to the lesions observed in histology. The clinical impact of these lesions is not easy to evaluate; their random localization prevented us from establishing a direct correlation with the aforementioned behavioral observations. Nonetheless, the present study highlighted that the irradiation did not prevent the pMBRT animals to correctly learn the tasks and to be as performing as control animals in the different cognitive evaluations.

## Conclusions

We performed the first comprehensive evaluation of the impact of pMBRT on brain functions. Our study revealed no significant dysfunction of motor or emotional processes. Regarding cognitive functions, no major deficits were observed in spatial learning, spatial memory, spatial strategy, associative learning or memory, decision making or temporal discrimination. Even though an occasional slight learning delay seems to be present in some tests, the pMBRT rats performed similarly to the control rats, suggesting the maintenance of the brain functions necessary for the successful completion of the tests. Besides, the histological lesions detected in the irradiated brains were minimal to moderate in most rats, and we could not correlate these lesions with a clinical impact. Collectively, our results suggest that pMBRT has a significantly lower impact on the brain tissue and functions than classical irradiation procedures.

Future studies will include functional evaluation of tumour-bearing animals after pMBRT.

## Supplementary information

Supplementary file1

## Data Availability

The datasets generated and analysed during the current study are available from the corresponding author on reasonable request.
