# Supplementary material for: Short and long-term evaluation of the impact of proton minibeam radiation therapy on motor, emotional and cognitive functions
Source: Sci Rep. 2020 Aug 11;10:13511. doi: 10.1038/s41598-020-70371-w (PMC7419511; doi:10.1038/s41598-020-70371-w)
Supplement: Supplementary file 1 — Supplementary file1 [file 41598_2020_70371_MOESM1_ESM.docx]

**Short and long-term evaluation of the impact of proton minibeam radiation therapy on motor, emotional and cognitive functions**

Charlotte Lamirault^1*^, Ph.D., Valérie Doyère^2^, Ph.D., Marjorie Juchaux^3^, Ph.D., Frederic Pouzoulet^1^, Ph.D., Dalila Labiod^1^, Ms, Remi Dendale^4^, MD, Ph.D., Annalisa Patriarca^4^, Ph.D., Catherine Nauraye^4^, Ph.D., Marine Le Dudal^5,6^, Ms, Grégory Jouvion^5,7^, Ph.D., David Hardy^5^, Ph.D., Nicole El Massioui^2^, Ph.D., and Yolanda Prezado^8*, **^, Ph.D.

^1^Institut Curie, PSL Research University, Translational Research Department, Experimental Radiotherapy Platform, Orsay, France

^2^Université Paris-Saclay, CNRS, Institut des Neurosciences Paris-Saclay, 91190, Gif-sur-Yvette, France.

^3^Laboratoire de Physique des 2 infinis Irène Joliot-Curie (IJCLab - UMR 9012) CNRS / Université Paris-Saclay / Université de Paris, Campus universitaire, Orsay, France

^4^Institut Curie, PSL Research University, Radiation Oncology Department, Centre de Protonthérapie d'Orsay, 101, F-91898 Orsay, France

^5^Institut Pasteur, Neuropathologie Expérimentale, 75015, Paris, France.

^6^Ecole Nationale Vétérinaire d’Alfort, Biopôle, Unité d’Histologie, d’Embryologie et d’Anatomie pathologique, Université Paris-Est, Maisons-Alfort, France

^7^Sorbonne Université, INSERM, Physiopathologie des Maladies Génétiques d’Expression Pédiatrique, Assistance Publique des Hôpitaux de Paris, Hôpital Armand-Trousseau, UF de Génétique Moléculaire, Paris, France

^8^Institut Curie, University Paris Saclay, PSL Research University, Inserm U 1021-CNRS UMR 3347, Orsay, France

*Previous affiliation : Laboratoire d’Imagerie et Modélisation en Neurobiologie et Cancérologie (IMNC), Centre National de la Recherche Scientifique (CNRS); Universités Paris-Saclay and Paris 7, Campus d’Orsay, 91405 Orsay, France.

** Corresponding author: Yolanda Prezado, Institut Curie, Bat 110, Campus \d’Orsay, France. Phone: +33169863059; Mail: yolanda.prezado@curie.fr

**Supplemental material**

**Detailed descriptions of test protocols**

Vertical grid test: The rats were hung upside down in the middle of a vertical grid (1.5 m distance above floor, 60 lux, and 60 db background noise in the experimental room). In this situation, the natural behavior of a rat is to turn around to go up the grid. The latency to body reversal (“return”) was measured to assess the motor coordination of rats.

Horizontal bar test: Each animal was hooked by its front paws to a horizontal metal bar (4 mm diameter, 60 cm distance above floor, 60 lux, and 60 db background noise in the experimental room) to measure the muscular tonus. The latency before the rat fell from the bar was recorded (maximum time=60 s).

Open field (OF) test: A square arena (1 m × 1 m × 50 cm height) was placed in a dimly lit room (30 lux in the center of the arena and 60 db background noise in the experimental room). Each rat was placed along one of the walls and could freely explore this environment. The rats usually spent their time in the peripheral zone (along the walls) rather than in the central zone to avoid exposure in the open arena, which may make them vulnerable to predators.

Motivation for food: This test assessed each animal’s motivation to consume food pellets. Each rat was placed individually in a closed arena (20 cm × 20 cm × 50 cm, 60 lux, and 60 db background noise in the experimental room) that had two small cups. One cup contained 5 g of normal pellets (45 mg “grain-based” TestDiet, Bio-concept Technologies), and the other contained 5 g of chocolate-flavored sucrose pellets (45 mg Chocolate Rodent Diet, Bio-Serv). The chocolate pellets were previously provided to the rats in their home cages in order to avoid neophobic bias and to ensure high motivational value.

Object recognition task (ORT) and object location task (OLT): The procedure consisted of three phases: (1) habituation phase: the OF test, which was conducted the day before, was considered habituation to the environment. (2) familiarization phase: the rats were placed in the arena (30 lux in the center of the arena and 60 db background noise in the experimental room) twice for 5 min (with an interval of 3 h) with two identical objects. (3) testing phase: 3 h later, the rats were placed in the arena for 3 min with one novel object (ORT) or with one object moved to a new location (OLT) and one familiar object and location. These tests were performed four times (48 h pre-irradiation and 1, 5, and 10 months after irradiation). The objects presented were always very different from one test to another in terms of shape, color, size, and material to prevent learning bias with the repetition of tests.

Morris water maze (MWM) test (14): For analysis, a pool (60 lux and 70 db white noise in experimental room) was divided into four quadrants (northwest [NW], southwest [SW], northeast [NE], and southeast [SE]); a platform was placed in NW for all acquisition trials. Each rat underwent four trials per day, with a 60 s trial limit. They had to find the platform (hidden 2 cm under the white-water surface) followed by a 60 s resting period (on the platform) before the next trial. Acquisition lasted for 9 days, amounting to 36 trials in total. Each day, the rats were released at four different starting positions randomized across days. Any rat that failed to find the platform within the time limit was led to the platform. The distance required to find the hidden platform was recorded. On the tenth day, a 60 s probe trial was performed with the platform removed, and distance and time spent in each quadrant were recorded.

Cross-maze test (15): This test was performed under food deprivation (rats maintained at 85% of their initial weight). A T-maze (30 lux and 60 db background noise in the experimental room) was created with three arms: east, west (at 180° to each other), and south (perpendicular to east and west). Two cups were present: one at the end of the east and west arms each, but only the east arm was baited (food pellets). Each rat started from the south arm and had to learn to go to the east arm to obtain the reward food pellets. Choosing the west arm was considered incorrect. The rats received four trials per day, and the number of correct responses (east arm choice) was measured.

Fear conditioning (16): Pavlovian fear conditioning consisted in pairing a conditioned stimulus (CS, a tone), which was initially neutral, with an aversive unconditioned stimulus (electrical foot shock). Therefore, a conditioned response of fear (CR, freezing) was induced by the presentation of the CS alone. On the first day, the animals were placed one by one in operant chamber (Coulbourn Instruments, USA; 34.3 × 34.3 × 50.8 cm ; 10 lux and 60 db background noise), where a tone (CS, 4 kHz, 80 dB, 30 s) was immediately followed by a short electrical foot shock (0.8 mA, 0.5 s). The session consisted of five tone–shock pairings separated by an inter-trial interval (ITI) of 4 min on average. During the next two days, each animal was placed in a new context (Context B, modified box [corners filled with a board to change the shape of the box], green ambient light, and peppermint odor) where 16 CS alone (not followed by foot shocks, i.e., in extinction) were delivered with an ITI of 2 min on average.

Rat gambling task (RGT) (17,18):

*Pretraining:* In operant chambers (Coulbourn Instruments, USA; 34.3 × 34.3 × 50.8 cm; 10 lux and 60 db background noise), each rat learned to associate two consecutive nose pokes in an illuminated hole with the delivery of one or two food pellets. This procedure continued daily until the rats achieved the criterion (80 or 160 pellets in less than 60 min).

*Gambling Task:* Each rat performed a test session (1 h) the day after the last training session. In this session, each animal could choose between the four simultaneously illuminated holes (A–D) and each choice was associated with a particular outcome. Choices A and B delivered immediately two food pellets but could be followed by long time-outs (no choice could be made), i.e. 222 s for the choice A and 444 s for the choice B, which was thus disadvantageous choices. The time-outs was delivered with a probability of 50% for the hole A and 25% for the hole B. By contrast, a single food pellet was delivered if the holes C and D were chosen but short time-outs could be also associate, i.e. 12 s for the choice C and 6 s for the choice D (advantageous choices) with a probability of 25% and 50% respectively. The position of advantageous and disadvantageous choices was counterbalanced within each group. At the end of the RGT session, the theoretical maximum gain was five times higher for the advantageous choices than for the disadvantageous choices.

Temporal discrimination (19):

*Training (19):* For 3 days, the animals were trained to press a lever associated with a tone of 2 s (right lever) and with a tone of 8 s (left lever) to obtain a food pellet. The sessions were composed of 100% forced-choice trials, i.e. only the correct lever (left or right) paired with the corresponding tone duration (2 or 8 s) was presented. During the 3 following days, sessions were composed of 50% forced-choice/50% free-choice trials. Finally, the sessions of the last 4 days were composed of 100% free-choice trials in which both levers were simultaneous presented. A session was composed of 80 trials (2 x 20 forced-choice/20 free choice or 2 x 40 free-choice) with a ITI of 30 s on average. The percentage of correct responses across all free-choice trials was measured.

*Bisection Tests (19):* A psychophysical choice session with the addition of five intermediate durations (2.5, 3.2, 4, 5, and 6.3 s, 12 trials each) was performed and only the correct responses to the anchor durations (2 and 8 s, 60 trials of each) were reinforced. The ITI was 20 s on average. The proportion of responses on the lever assigned as correct for the long-duration stimulus on all trials with a response was measured. For all trials with a response, the proportion of “long” responses to stimulus duration were averaged across sessions. Then, a pseudo logistic model fit (Prism, GraphPad Software) was used to analyze these bisection functions. The stimulus value corresponding to p(long) = 0.5 was used to determine the point of subjective equality (PSE). For each rat, gamma was also determined as proportional to the Weber fraction and which is inversely related to temporal sensitivity.

Histopathological assessment:

| Levels of section | 1 | 2 | 3 | 4 |
| --- | --- | --- | --- | --- |
| Control  (left and right brain) | H&E, IBA-1, GFAP, Act. Casp. 3, LFB | / | / | H&E, IBA-1, Act. Casp. 3 |
| pMBRT  (left and right brain) | H&E, IBA-1, GFAP, Act. Casp. 3, LFB | H&E, IBA-1 | H&E, IBA-1 | H&E, IBA-1, Act. Casp. 3 |

**Table 1.** For the histopathological analysis, we carried out sagittal sections at different levels in the left and right parts of the brain, for every rat. For the irradiated group, we carried out 4 levels (separated by at least 200 micron). The analyses performed at each section level are described in the table. For the control group, we carried out 2 levels (separated by at least 600 micron). The analyses performed at each section level are also described in the table.

HE (Hematoxylin and Eosin) staining to describe the lesions, Iba-1 (Ionized calcium-binding adapter molecule 1; marker of microglial cells), GFAP (Glial Fibrillary Acidic Protein; marker of astrocytes), Act. Casp. 3 (Activated Caspase 3; marker of apoptosis), LFB (Luxol Fast Blue; staining of myelin).

**
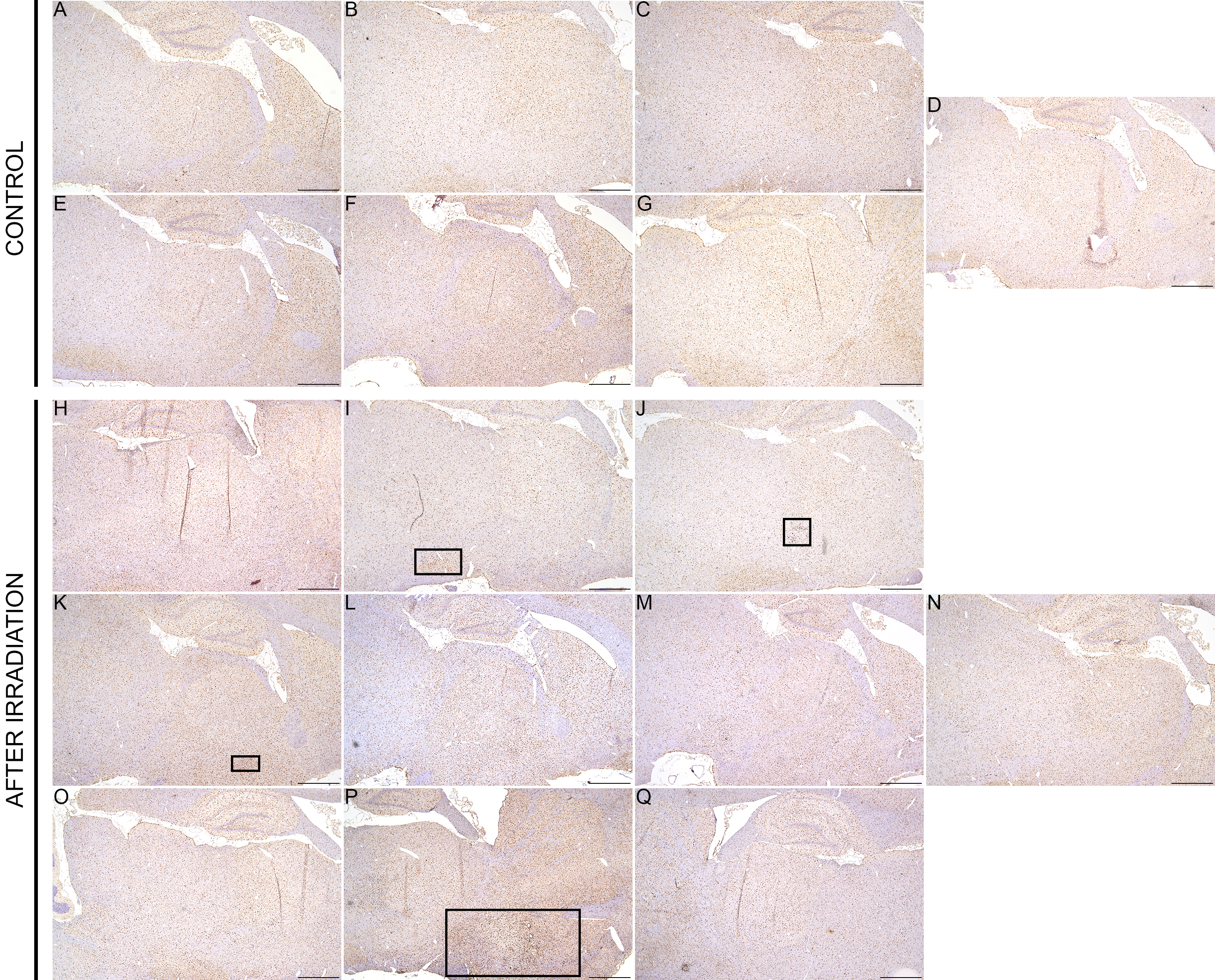
**

**Figure 7.** Low magnification (Iba1 immunohistochemistry) of sagittal brain sections (scale bar: 1 mm) of each rat showing small loci of reactive microglial cells in three animals (I, J, K), after irradiation (black squares). Only one rat (P) displayed more extensive/severe lesions.
